# Inferior Epigastric Artery Pseudoaneurysm in a Kidney Transplant Recipient

**DOI:** 10.1155/2013/459320

**Published:** 2013-07-22

**Authors:** M. Tozzi, B. Molteni, M. Franchin, G. Ietto, G. Soldini, V. Bertocchi, G. Carcano

**Affiliations:** ^1^Vascular Surgery, Insubria University, Circolo University Teaching Hospital, Via Guicciardini 9, 21100 Varese, Italy; ^2^Department of Organ Transplant, Insubria University, Circolo University Teaching Hospital, Via Guicciardini 9, 21100 Varese, Italy

## Abstract

Pseudoaneurysm of inferior epigastric artery (IEA) is a very rare clinical entity. We reported a case of combined kidney transplant and pseudoaneurysmectomy in a young HBV-HCV-HIV recipient. This case emphasizes the possibility of planning a safe and correct surgical treatment and the best timing to treat IEA pseudoaneurysm. An exhaustive preoperative radiological study in all patients candidate to kidney transplant could identify the possible aortoiliac disease both stenotic or dilatative even if it is rare and helps to define the best treatment options.

## 1. Introduction

Pseudoaneurysm of inferior epigastric artery (IEA) is a very rare clinical entity, with just a few cases reported in the literature. It is described as a complication of surgery, trauma, arterial puncture, paracentesis, and removal of Tenckhoff catheters and sometimes is idiopathic. 

IEA pseudoaneurysm can be diagnosed by a contrast enhanced computer tomography (CT) scan or by a color Doppler ultrasound (US). 

The treatment options include surgery with excision and ligation of IEA, percutaneous embolization with placement of metallic coils or with N-butyl cyanoacrylate (NCBA), percutaneous thrombin injection, sonographic-guided compression, and conservative treatment [1–25].

## 2. Case Report

**Figure 1:**
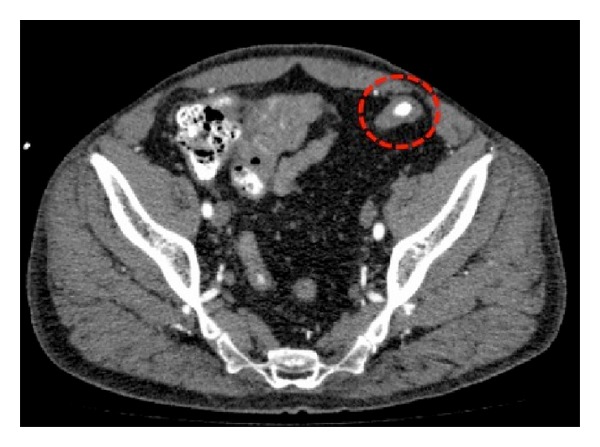


**Figure 2:**
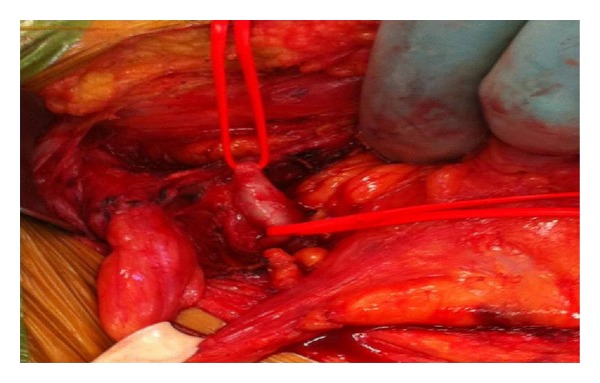


A 51-year-old male marble cutter, with chronic hepatopathy HBV-HCV related, HIV infection, hypertension, and chronic obstructive pulmonary disease, developed end-stage HIV nephropathy. The patient had no prior surgical treatment. The patient started dialysis in 2009. In 2010, he was considered for kidney transplant. In consideration of comorbidities, angiographic-CT scan was performed and detected a 8 mm pseudoaneurysm of left IEA close to the vassel origin (Figure 1). The patient did not refer to pain or discomfort; no tender mass was detected at palpatory examination. Kidney transplant was performed approximately 20 months after trough a “hockey stick” incision; renal graft was anastomized to common-iliac vessels. Contemporary IEA ligation at vassel origin and pseudoaneurymectomy were performed (Figure 2). Postoperative course was regular. During hospitalization, a renal tract US was performed in 4th and 10th postoperative days and no perigraft or pericystic fluid collection was detected. Hospitalization time was 12 days and at the discharge creatinine serical level was 1,4 mg/dL, haemoglobin 13,6 g/dL. At 1 month, creatinine level was 1.2 mg/dL. 

## 3. Discussion

The IEA arises as a branch of the external iliac artery just above the inguinal ligament. From its origin, it courses upward and medially to enter the rectus sheath. Within the rectus sheath, it lies just at anterior to posterior wall of the sheath [1]. Pseudoaneurysm of the IEA is a recognised but very rare clinical entity with just a few cases reported in the literature as complication of surgery, trauma, and arterial puncture [2–4]. Other very unusual causes are sutures, drain-tube or Tenckoff catheters insertion, therapeutic paracentesis, manipulation of laparoscopic instruments [26], and abdominal wall surgery [19]; only 3 cases of idiopathic IEA pseudoaneurysm were described in the literature [11]. An IEA pseudoaneurysm and arteriovenous fistula as a complication of IEA surgical injury are reported [13].

 IEA pseudoaneurysm could be treated by feeding vessel surgical ligation and aneurysmectomy [2, 4–7, 9, 10, 12, 25], by percutaneous coil embolisation [3, 8, 13–15, 17–19, 22, 24], by ultrasound guided thrombin injection, by ultrasound guided compression, or by conservative treatment with a spontaneous regression [16, 20, 23]. Surgery is the most invasive treatment and includes the necessity of general anaesthesia and postoperative pain treatment, but the advantage is the quick resolution of the symptoms with the removal of the mass effect caused by the pseudoaneurysm [18]. Ferrer et al. recommended surgery for large pseudoaneurysm [5]. Percutaneous embolization is generally used, but the disadvantages of percutaneous transcatheter approach for the management of IEA pseudoaneurysms include the necessity of arterial access, sheath placement, and increased potential for femoral pseudoaneurysm (0,8–2,2%) [27]. Lam et al. suggested that percutaneous transcatheter techniques may be preferable in patients with portal hypertension caused by chronic liver disease, who often have a coagulopathy and increased venous collateral in their abdominal wall [8]. Embolization with metallic coils or microcoils is successful, but it depends on the anatomy, the size of the arterial lesion, and the technical ability to place selectivity or superselectivity catheters or microcatheters [1]; also NBCA has been successfully used to treat IEA pseudoaneurysm [24]. US-guided probe compression may be useful for initial management of the pseudoaneurysms, but it may not be adequate if the pseudoaneurysm is deep seated either secondary to a large haematoma or it has a wide neck [15]; pain and discomfort at the compression site, the longtime compression (30–50 min), and incomplete occlusion constitute drawbacks [3, 4]. Ultrasonographic-guided thrombin injection is simple, safe, and quick to perform, painless and extremely effective, but the risk of thrombosis of patent artery because of the leakage of thrombin into the supply artery should be considered [1]. Spontaneous thrombosis has been reported after conservative management; the authors believe that the rise of intracompartimental pressure caused by hematoma was enough to achieve thrombosis [23].

Clinically, IEA pseudoaneurysms are difficult to diagnose because they usually present as diffuse tender masses that are not pulsatile. They are therefore difficult to differentiate from simple haematomas, which can also present as tender masses [1]. Epigastric vassels are usually located in the area between 4 and 8 cm from the midline [28]; clinical suspicions for an IEA pseudoaneurysm should be raised when any patient presents with a painful abdominal wall mass <8 cm from either side of midline whether or not is found to be pulsatile [18]. Because of the communication with the injured artery, an audible bruit may sometimes be present over the pseudoaneurysm [2, 6, 26], which excludes a hematoma.

Diagnosis of IEA pseudoaneurysms is usually by a contrast-enhanced CT scan or a color Doppler US with the latter being the imaging modality of choice [9, 11–13, 15]. Dopple US has a sensitivity and specificity of 100% in the differentiation of pseudoaneurysms from periarterial hematoma [29] and shows the classic “to-and-fro” sign of a pseudoaneurysm, which is caused by flow change within the pseudoaneurysm during the systole and diastole [1].

## 4. Conclusion

IEA pseudoaneurysm surgical treatment is a good choice if it can be performed contextually with another surgical laparotomy as in kidney transplant. That implies that the disadvantages of surgery, as general anesthesia and postoperative pain, are reduced from 2 times to just 1 time. In addition, during kidney transplantation, ligation of IEA is usually performed; therefore in our patient surgical treatment contextually with the kidney transplant was the best choice even if pseudoaneurysm size was small. In our patient an exhaustive radiological imaging study was important in order to detect possible vascular abnormalities in kidney recipient prior to transplantation, that give us the opportunity to detect IEA pseudoaneurysm. The literature largely demonstrates how physical examination alone cannot be always sufficient to detect aortoiliac district damage either in stenotic or dilatative way. In our experience, abdominal contrast-enhanced CT or Doppler study is mandatory for all patients waiting a kidney transplant with two or more risk factors for vasculopathies or elder than 55 years in order to plan and timing the correct surgical treatment of vascular lesions. Preoperative studies can also help to detect rare vascular lesions and give the opportunity to plan the correct treatment and timing, as in this case report during the kidney transplant laparotomy.

## Abbreviations and Acronyms

CT: Computer tomography
IEA: Inferior epigastric artery
NBCA: n-Butyl cyanoacrylate
US: Ultrasound.
